# The genome sequence of the sea mat, *Membranipora membranacea* (Linnaeus, 1767)

**DOI:** 10.12688/wellcomeopenres.18855.1

**Published:** 2023-01-24

**Authors:** John Bishop, Patrick Adkins, Christine Wood, Helen Jenkins, Marine Biological Association Genome Acquisition Lab, Darwin Tree of Life Barcoding collective, Wellcome Sanger Institute Tree of Life programme, Wellcome Sanger Institute Scientific Operations: DNA Pipelines collective, Tree of Life Core Informatics collective, Darwin Tree of Life Consortium

**Affiliations:** 1 Marine Biological Association, Plymouth, Devon, UK

**Keywords:** Membranipora membranacea, sea mat, genome sequence, chromosomal, Bryozoa

## Abstract

We present a genome assembly from an adult colony of *Membranipora membranacea* (the sea mat; Bryozoa; Gymnolaemata; Cheilostomatida; Membraniporidae). The genome sequence is 339 megabases in span. Most of the assembly (99.95%) is scaffolded into 11 chromosomal pseudomolecules. The mitochondrial genome has also been assembled and is 14.7 kilobases in length.

## Species taxonomy

Eukaryota; Metazoa; Spiralia; Lophotrochozoa; Bryozoa; Gymnolaemata; Cheilostomatida; Malacostegina; Membraniporoidea; Membraniporidae; *Membranipora*; *Membranipora membranacea* (Linnaeus, 1767) (NCBI:txid95170).

## Background

The bryozoan *Membranipora membranacea* is most often encountered as broad, white, lacy patches on kelp blades cast up on the shore. This species is a kelp specialist, rarely growing on anything else, and the calcified zooid walls have flexible hinges that allow the colony to flex with the kelp. Colony growth on Laminaria kelps is directed towards the frond’s base, accessing the region most likely to survive the winter ( Lutaud, 1961; Ryland & Stebbing, 1971). Large colonies can expand by as much as 10 mm a day ( Lutaud, 1961), with numerous generations of partially formed zooids in a pale growth zone at the colony edge. Colonization by *M. membranacea* as a non-native species on the northeastern coast of North America makes the local kelps more susceptible to storm breakage. This produces gaps in kelp canopies, facilitating invasion by non-native algae ( Scheibling & Gagnon, 2006).

*Membranipora membranacea* releases eggs that develop into a planktotrophic ‘cyphonautes’ larva, flattened between two triangular shells, rather than the non-feeding, shell-less ‘coronate’ larva that is brooded by most present-day bryozoans. Unusually, the settled larva metamorphoses to produce twin primary zooids, rather than a single zooid. The species is a member of a grouping referred to as malacostegan cheilostomatids, all sharing a naked membranous upper surface to the zooids and development as a cyphonautes larva. The malacostegans are paraphyletic to the inclusion of all other cheilostomatids, and include the earliest-branching cheilostomatid species in the molecular phylogeny of ( Waeschenbach *et al.*, 2012). Large *M. membranacea* colonies can provide huge numbers of embryos for experimental work. These have been used to investigate the molecular identity and fate of the early blastomeres in the distinctive biradial pattern of cleavage seen in bryozoans, and relate this to the spiralian cleavage pattern that is highly conserved in many other invertebrate groups ( Vellutini *et al.*, 2017). 

The genome of the sea mat, *M. membranacea*, was sequenced as part of the Darwin Tree of Life Project, a collaborative effort to sequence all named eukaryotic species in the Atlantic Archipelago of Britain and Ireland.

## Genome sequence report

**Figure 1.**
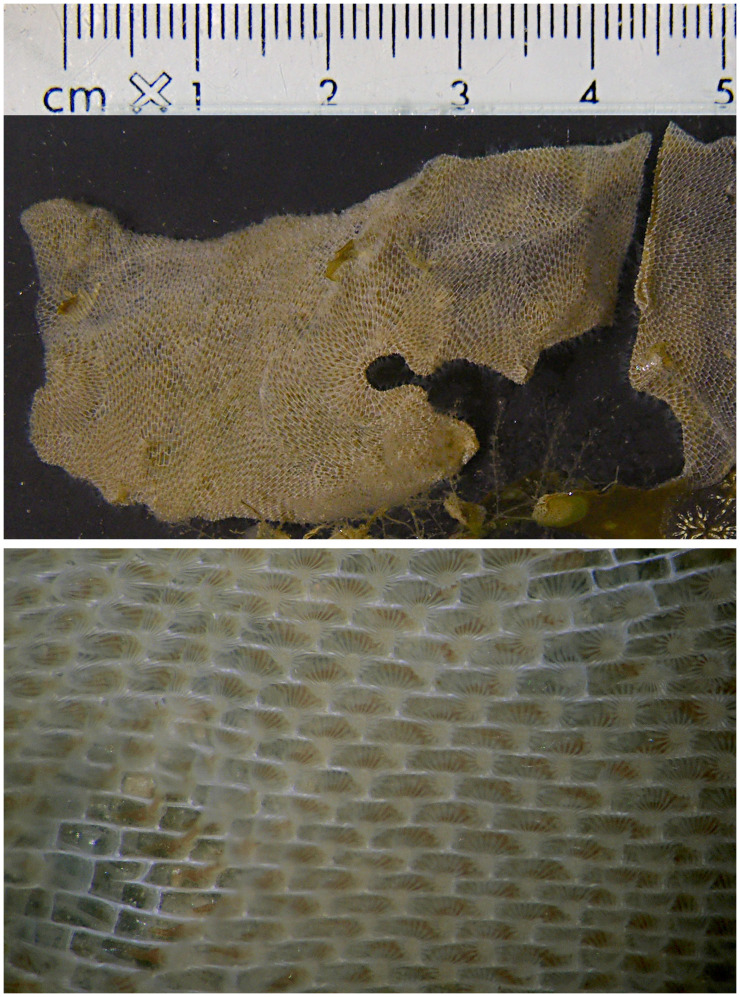
Photographs of the *Membranipora membranacea* (tzMemMemb1) specimen used for genome sequencing. The upper panel shows part of the colony (the cm scale refers to this) and the lower panel shows a close-up showing an array of zooids feeding with their extended lophophores.

The genome was sequenced from a *M. membranacea* colony ( Figure 1) collected from Queen Anne’s Battery Marina visitors’ pontoon, Plymouth (50.36, –4.13). A total of 66-fold coverage in Pacific Biosciences single-molecule HiFi long reads and 173-fold coverage in 10X Genomics read clouds were generated. Primary assembly contigs were scaffolded with chromosome conformation Hi-C data. Manual assembly curation corrected 62 missing or mis-joins and removed 17 haplotypic duplications, reducing the assembly length by 1.94% and the scaffold number by 79.81%, and increasing the scaffold N50 by 1.73%.

**Figure 2.**
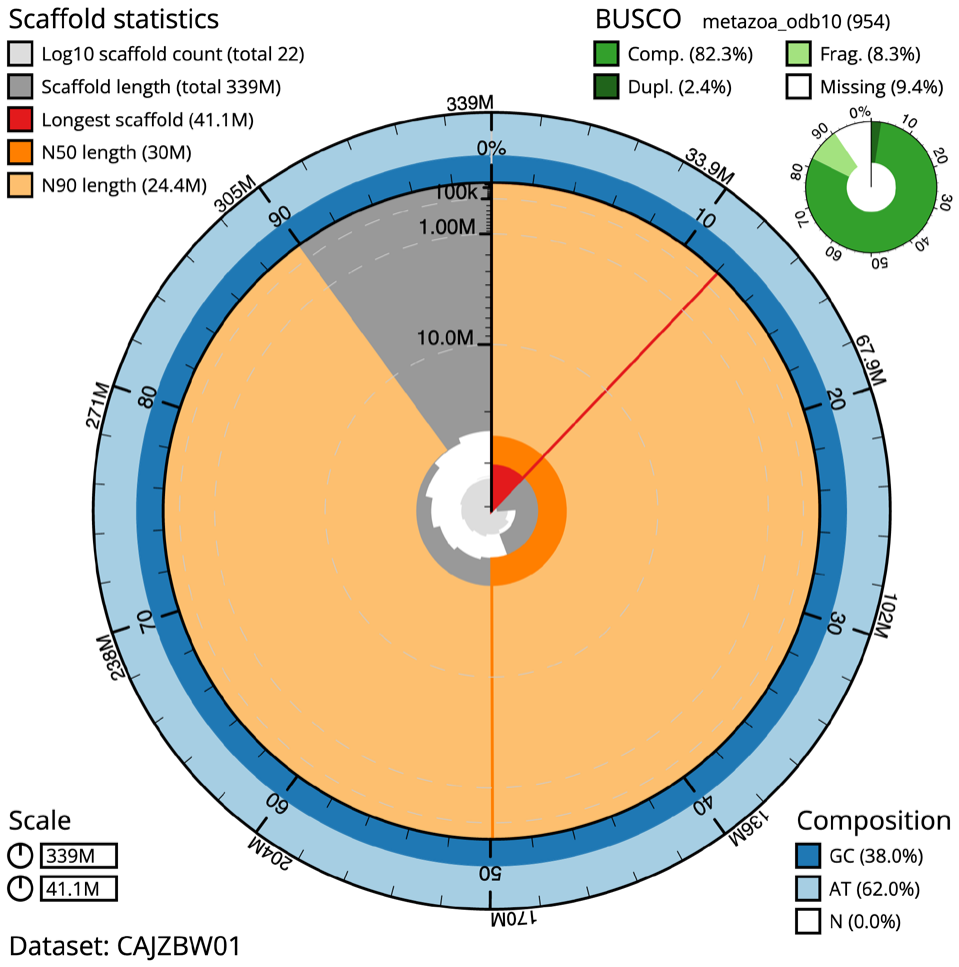
Genome assembly of *Membranipora membranacea*, tzMemMemb1.1: metrics. The BlobToolKit Snailplot shows N50 metrics and BUSCO gene completeness. The main plot is divided into 1,000 size-ordered bins around the circumference with each bin representing 0.1% of the 339,374,674 bp assembly. The distribution of scaffold lengths is shown in dark grey with the plot radius scaled to the longest scaffold present in the assembly (41,072,641 bp, shown in red). Orange and pale-orange arcs show the N50 and N90 scaffold lengths (29,963,023 and 24,422,204 bp), respectively. The pale grey spiral shows the cumulative scaffold count on a log scale with white scale lines showing successive orders of magnitude. The blue and pale-blue area around the outside of the plot shows the distribution of GC, AT and N percentages in the same bins as the inner plot. A summary of complete, fragmented, duplicated and missing BUSCO genes in the metazoa_odb10 set is shown in the top right. An interactive version of this figure is available at https://blobtoolkit.genomehubs.org/view/tzMemMemb1.1/dataset/CAJZBW01/snail.

The final assembly has a total length of 339.4 Mb in 21 sequence scaffolds with a scaffold N50 of 30.0 Mb ( Table 1). Most (99.95%) of the assembly sequence was assigned to 11 chromosomal-level scaffolds. Chromosome-scale scaffolds confirmed by the Hi-C data are named in order of size ( Figure 2– Figure 5; Table 2). While not fully phased, the assembly deposited is of one haplotype. Contigs corresponding to the second haplotype have also been deposited. The assembly has a BUSCO v5.1.2 ( Manni *et al.*, 2021) completeness of 82.3% (single 79.9%, duplicated 2.4%) using the OrthoDB-v10 metazoa reference set. BUSCO loci identified as fragmented accounted for a further 8.3% of loci tested. The low BUSCO score may be due to low conservation of orthologues between *M. membranacea* and the metazoan species in the reference set, or underperformance of the BUSCO gene finder, given the particular gene structures in this species.

**Table 1.** Genome data for *Membranipora membranacea*, *tzMemMemb1.1*.

Project accession data		
Assembly identifier	tzMemMemb1.1	
Species	*Membranipora membranacea*	
Specimen	tzMemMemb1	
NCBI taxonomy ID	95170	
BioProject	PRJEB45195	
BioSample ID	SAMEA7536681	
Isolate information	modular colony	
Assembly metrics *		*Benchmark*
BUSCO **	C:82.3%[S:79.9%,D:2.4%], F:8.3%,M:9.4%,n:954	*C ≥ 95%*
Percentage of assembly mapped to chromosomes	99.95%	*≥ 95%*
Organelles	mitochondrial genome assembled	*complete single alleles*
Raw data accessions		
PacificBiosciences SEQUEL II	ERR6406215	
10X Genomics Illumina	ERR6054950–ERR6054953	
Hi-C Illumina	ERR6054954	
PolyA RNA-Seq Illumina	ERR6464929	
Genome assembly		
Assembly accession	GCA_914767715.1	
*Accession of alternate haplotype*	GCA_914767555.1	
Span (Mb)	339.4	
Number of contigs	93	
Contig N50 length (Mb)	7.0	
Number of scaffolds	21	
Scaffold N50 length (Mb)	30.0	
Longest scaffold (Mb)	41.1	

* Assembly metric benchmarks are adapted from column VGP-2020 of “Table 1: Proposed standards and metrics for defining genome assembly quality” from ( Rhie *et al.*, 2021).

** BUSCO scores based on the metazoa_odb10 BUSCO set using v5.1.2. C = complete [S = single copy, D = duplicated], F = fragmented, M = missing, n = number of orthologues in comparison. A full set of BUSCO scores is available at https://blobtoolkit.genomehubs.org/view/tzMemMemb1.1/dataset/CAJZBW01/busco.

**Figure 3.**
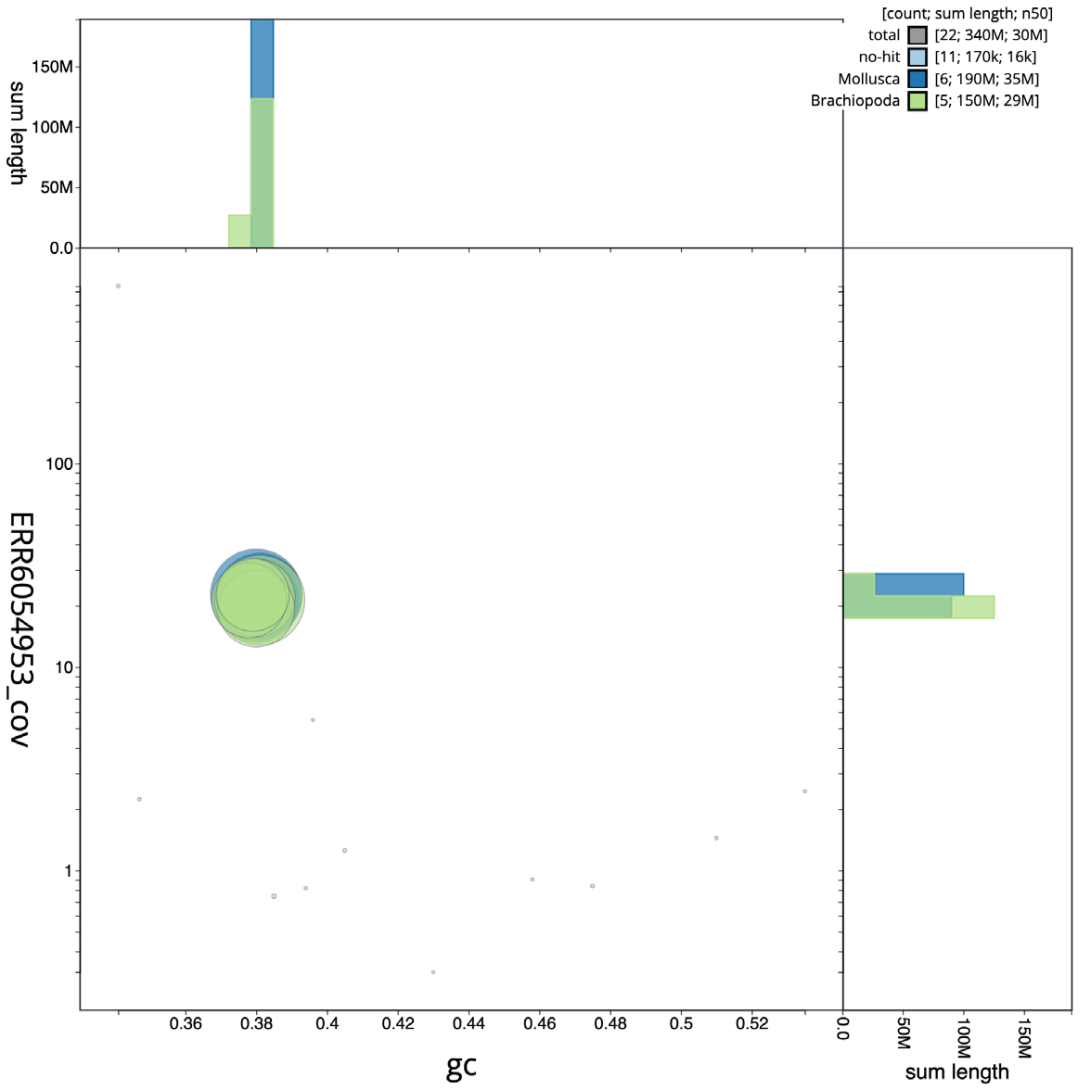
Genome assembly of *Membranipora membranacea*, tzMemMemb1.1: GC coverage. BlobToolKit GC-coverage plot. Scaffolds are coloured by phylum. Circles are sized in proportion to scaffold length. Histograms show the distribution of scaffold length sum along each axis. An interactive version of this figure is available at https://blobtoolkit.genomehubs.org/view/tzMemMemb1.1/dataset/CAJZBW01/blob.

**Figure 4.**
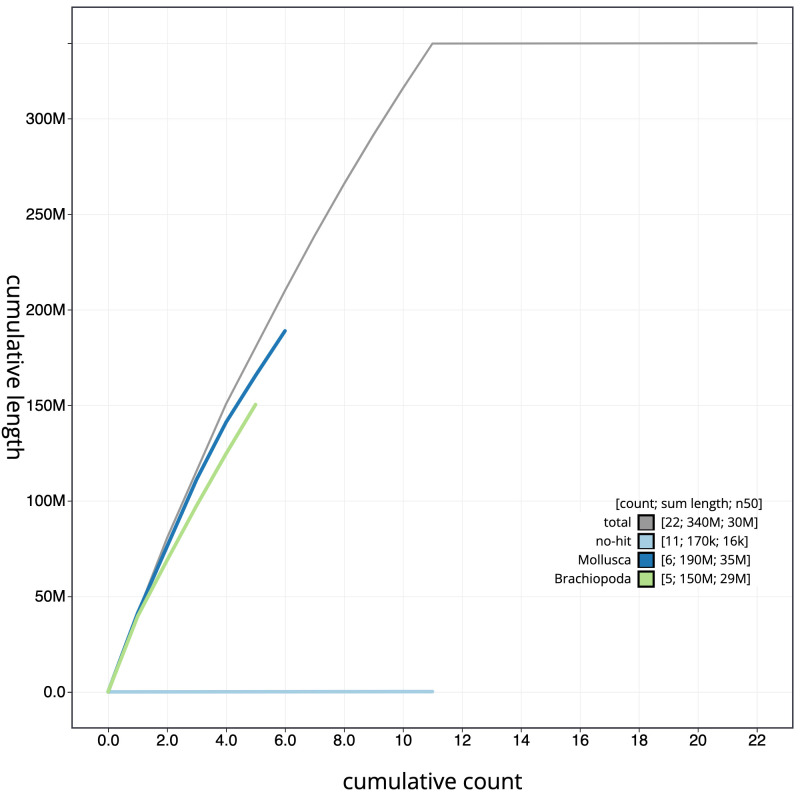
Genome assembly of *Membranipora membranacea*, tzMemMemb1.1: cumulative sequence. BlobToolKit cumulative sequence plot. The grey line shows cumulative length for all scaffolds. Coloured lines show cumulative lengths of scaffolds assigned to each phylum using the buscogenes taxrule. An interactive version of this figure is available at https://blobtoolkit.genomehubs.org/view/tzMemMemb1.1/dataset/CAJZBW01/cumulative.

**Figure 5.**
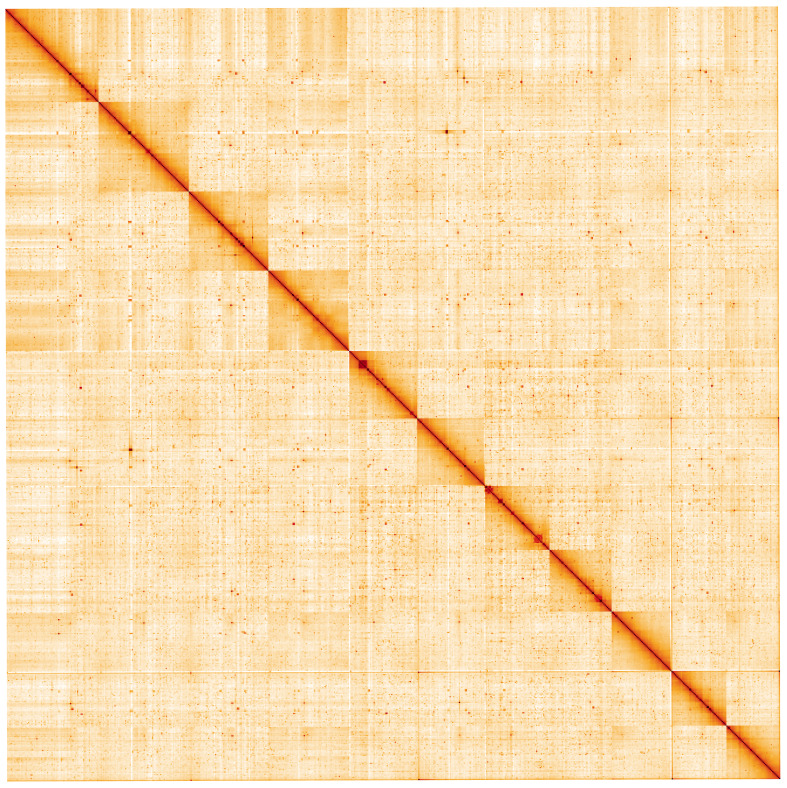
Genome assembly of *Membranipora membranacea*, tzMemMemb1.1: Hi-C contact map. Hi-C contact map of the tzMemMemb1.1 assembly, visualised using HiGlass. Chromosomes are shown in order of size from left to right and top to bottom. An interactive version of this figure may be viewed at https://genome-note-higlass.tol.sanger.ac.uk/l/?d=HIms5gzmQMSsRCruPkDsiQ.

**Table 2.** Chromosomal pseudomolecules in the genome assembly of *Membranipora membranacea*, *tzMemMemb1*.

INSDC	Chromosome	Size (Mb)	GC%
OU612065.1	1	41.07	38
OU612066.1	2	39.53	38.1
OU612067.1	3	35.04	38
OU612068.1	4	35	38.1
OU612069.1	5	29.96	38
OU612070.1	6	29.39	38
OU612071.1	7	28.51	38
OU612072.1	8	27.17	37.8
OU612073.1	9	25.74	37.9
OU612074.1	10	24.42	38
OU612075.1	11	23.37	38.1
OU612076.1	MT	0.01	34
-	-	0.15	42

## Methods

### Sample acquisition and nucleic acid extraction

A *M. membranacea* (tzMemMemb1) colony was collected from Queen Anne’s Battery Marina visitors’ pontoon, Plymouth, UK (latitude 50.36, longitude –4.13) on 15 July 2020. The specimen, a colony growing on *Undaria pinnatifida*, was collected by hand and preserved in liquid nitrogen. The collectors and identifiers were Patrick Adkins, John Bishop, Helen Jenkins and Chris Wood (Marine Biological Association).

DNA was extracted at the Tree of Life laboratory, Wellcome Sanger Institute. The tzMemMemb1 sample was weighed and dissected on dry ice with tissue set aside for Hi-C sequencing. The tissue was cryogenically disrupted to a fine powder using a Covaris cryoPREP Automated Dry Pulveriser, receiving multiple impacts. High molecular weight (HMW) DNA was extracted using the Qiagen MagAttract HMW DNA extraction kit. Low molecular weight DNA was removed from a 20 ng aliquot of extracted DNA using 0.8X AMpure XP purification kit prior to 10X Chromium sequencing; a minimum of 50 ng DNA was submitted for 10X sequencing. HMW DNA was sheared into an average fragment size of 12–20 kb in a Megaruptor 3 system with speed setting 30. Sheared DNA was purified by solid-phase reversible immobilisation using AMPure PB beads with a 1.8X ratio of beads to sample to remove the shorter fragments and concentrate the DNA sample. The concentration of the sheared and purified DNA was assessed using a Nanodrop spectrophotometer and Qubit Fluorometer and Qubit dsDNA High Sensitivity Assay kit. Fragment size distribution was evaluated by running the sample on the FemtoPulse system.

RNA was extracted from tissue of tzMemMemb1 in the Tree of Life Laboratory at the WSI using TRIzol, according to the manufacturer’s instructions. RNA was then eluted in 50 μl RNAse-free water and its concentration assessed using a Nanodrop spectrophotometer and Qubit Fluorometer using the Qubit RNA Broad-Range (BR) Assay kit. Analysis of the integrity of the RNA was done using Agilent RNA 6000 Pico Kit and Eukaryotic Total RNA assay.

### Sequencing

Pacific Biosciences HiFi circular consensus and 10X Genomics read cloud DNA sequencing libraries were constructed according to the manufacturers’ instructions. Poly(A) RNA-Seq libraries were constructed using the NEB Ultra II RNA Library Prep kit. DNA and RNA sequencing was performed by the Scientific Operations core at the WSI on Pacific Biosciences SEQUEL II (HiFi) Illumina HiSeq 4000 (RNA-Seq) and NovaSeq 6000 (10X) instruments. Hi-C data were also generated from the same specimen using the Arima v2 kit and sequenced on the Illumina NovaSeq 6000 instrument.

### Genome assembly

Assembly was carried out with Hifiasm ( Cheng *et al.*, 2021) and haplotypic duplication was identified and removed with purge_dups ( Guan *et al.*, 2020). One round of polishing was performed by aligning 10X Genomics read data to the assembly with Long Ranger ALIGN, calling variants with freebayes ( Garrison & Marth, 2012). The assembly was then scaffolded with Hi-C data ( Rao *et al.*, 2014) using SALSA2 ( Ghurye *et al.*, 2019). The assembly was checked for contamination and corrected using the gEVAL system ( Chow *et al.*, 2016) as described previously ( Howe *et al.*, 2021). Manual curation was performed using gEVAL, HiGlass ( Kerpedjiev *et al.*, 2018) and Pretext ( Harry, 2022). The mitochondrial genome was assembled using MitoHiFi ( Uliano-Silva *et al.*, 2022), which performed annotation using MitoFinder ( Allio *et al.*, 2020). The genome was analysed and BUSCO scores generated within the BlobToolKit environment ( Challis *et al.*, 2020). Table 3 contains a list of all software tool versions used, where appropriate.

**Table 3.** Software tools and versions used.

Software tool	Version	Source
BlobToolKit	2.6.2	Challis *et al.*, 2020
freebayes	1.3.1-17-gaa2ace8	Garrison & Marth, 2012
gEVAL	N/A	Chow *et al.*, 2016
Hifiasm	0.12	Cheng *et al.*, 2021
HiGlass	1.11.6	Kerpedjiev *et al.*, 2018
Long Ranger ALIGN	2.2.2	https:// support.10xgenomics. com/genome-exome/ software/pipelines/latest/ advanced/other-pipelines
MitoHiFi	2	Uliano-Silva *et al.*, 2022
PretextView	0.2	Harry, 2022
purge_dups	1.2.3	Guan *et al.*, 2020
SALSA	2.2	Ghurye *et al.*, 2019

### Ethics/compliance issues

The materials that have contributed to this genome note have been supplied by a Darwin Tree of Life Partner. The submission of materials by a Darwin Tree of Life Partner is subject to the Darwin Tree of Life Project Sampling Code of Practice. By agreeing with and signing up to the Sampling Code of Practice, the Darwin Tree of Life Partner agrees they will meet the legal and ethical requirements and standards set out within this document in respect of all samples acquired for, and supplied to, the Darwin Tree of Life Project. Each transfer of samples is further undertaken according to a Research Collaboration Agreement or Material Transfer Agreement entered into by the Darwin Tree of Life Partner, Genome Research Limited (operating as the Wellcome Sanger Institute), and in some circumstances other Darwin Tree of Life collaborators.

## Data Availability

European Nucleotide Archive: *Membranipora membranacea* (sea mat). Accession number PRJEB45195; https://identifiers.org/ena.embl/PRJEB45195 ( Wellcome Sanger Institute, 2021). The genome sequence is released openly for reuse. The *M. membranacea* genome sequencing initiative is part of the Darwin Tree of Life (DToL) project. All raw sequence data and the assembly have been deposited in INSDC databases. The genome will be annotated using available RNA-Seq data and presented through the Ensembl pipeline at the European Bioinformatics Institute. Raw data and assembly accession identifiers are reported in Table 1. Members of the Marine Biological Association Genome Acquisition Lab are listed here: https://doi.org/10.5281/zenodo.4783605. Members of the Darwin Tree of Life Barcoding collective are listed here: https://doi.org/10.5281/zenodo.4893703. Members of the Wellcome Sanger Institute Tree of Life programme are listed here: https://doi.org/10.5281/zenodo.4783585. Members of Wellcome Sanger Institute Scientific Operations: DNA Pipelines collective are listed here: https://doi.org/10.5281/zenodo.4790455. Members of the Tree of Life Core Informatics collective are listed here: https://doi.org/10.5281/zenodo.5013541. Members of the Darwin Tree of Life Consortium are listed here: https://doi.org/10.5281/zenodo.4783558.
