# Supplementary material for: The genome sequence of the sea mat, Membranipora membranacea (Linnaeus, 1767)
Source: Wellcome Open Res. 2023 Jan 24;8:38. [Version 1] doi: 10.12688/wellcomeopenres.18855.1 (PMC10290214; doi:10.12688/wellcomeopenres.18855.1)
Supplement: Peer review history

# Peer review: Reviewer response for version 1

**DOI:** 10.21956/wellcomeopenres.20907.r57273

**Published:** 2023-06-23

**Authors:** Chenhong Li

**Affiliations:** 1 Shanghai Universities Key Laboratory of Marine Animal Taxonomy and Evolution, Shanghai Ocean University, Shanghai, China

The data are sufficient and well analyzed.

 The low BUSCO score probably was due to low conservation of orthologues between *M. membranacea* and the metazoan species as suggested by the authors. Can other alternative tools be used for testing that? 

 In data availability, it says "The genome will be annotated using available RNA-Seq data and presented through the Ensembl pipeline at the European Bioinformatics Institute". Can you provide a link to it?

Are sufficient details of methods and materials provided to allow replication by others?

Yes

Is the rationale for creating the dataset(s) clearly described?

Yes

Are the datasets clearly presented in a useable and accessible format?

Yes

Are the protocols appropriate and is the work technically sound?

Yes

Reviewer Expertise:

Fish genomes

I confirm that I have read this submission and believe that I have an appropriate level of expertise to confirm that it is of an acceptable scientific standard.

# Peer review: Reviewer response for version 1

**DOI:** 10.21956/wellcomeopenres.20907.r57274

**Published:** 2023-05-23

**Authors:** Nansheng Chen

**Affiliations:** 1 Simon Fraser University, Burnaby, British Columbia, Canada

This manuscript described genome sequences of the sea mat, *Membranipora membranacea* for the first time, as part of the Darwin Tree of Life Project. 

 There are some minor issues:

1. Figure 1, lower panel does not have a scale. 
2. The sentence "The malacostegans are paraphyletic to the inclusion of all other cheilostomatids, and include the earliest-branching cheilostomatid species in the molecular phylogeny of " is truncated. 
3. The BUSCO results based on eukaryota_odb10 are far better than that based on metazoa_odb10. The authors should discuss. 
4. Figure 2 displays GC content and AT content along the entire genome. The GC and AT content curves were perfectly smooth. Does this mean that the GC contents do not change along the genome? 

Are sufficient details of methods and materials provided to allow replication by others?

Yes

Is the rationale for creating the dataset(s) clearly described?

Yes

Are the datasets clearly presented in a useable and accessible format?

Yes

Are the protocols appropriate and is the work technically sound?

Yes

Reviewer Expertise:

Comparative genomics, algorithm development

I confirm that I have read this submission and believe that I have an appropriate level of expertise to confirm that it is of an acceptable scientific standard.

# Peer review: Reviewer response for version 1

**DOI:** 10.21956/wellcomeopenres.20907.r54496

**Published:** 2023-02-07

**Authors:** Andreas Hejnol, Lisa-Marie Barf

**Affiliations:** 1 Friedrich Schiller University Jena, Jena, Germany; 2 University of Bergen, Bergen, Norway

This paper presents the genome assembly of the species *Membranipora membranacea* (Linnaeus, 1767), commonly known as sea mat, in 11 chromosomal pseudomolecules.

 The paper uses state-of-the-art sequencing methods, as well as suitable bioinformatic tools for eukaryote genome assembly. All data is made available and referenced under the respective accession ID.

 The generated RNA-Seq data were not analyzed in the scope of the paper but are made available for future studies such as genome annotation. Overall the work of the paper contributes to the completion of sequencing 70.000 eukaryotic genomes as intended by the Darwin Tree of Life project. It will be a valuable resource for future research making it possible to include the genome of the species *Membranipora membranacea*.

**Minor revisions:**

- Throughout the manuscript, the contents of the assembly are referred to as “scaffolds”, “chromosome-like scaffolds” and “chromosomal pseudomolecules”. It might be better to use a consistent description.
- Looking at the heatmap in Figure 5 there is a legend present in the interactive version which is missing in the manuscript. Please consider adding the legend to Figure 5 in the manuscript as well.
- The manuscript mentions the assembly of the mitochondrial genome. It would be interesting to include and elaborate further on the completeness of the mitochondrial genome. 
- What part of the animal was used to extract DNA and RNA, respectively? This is not described clearly in the methods. Different parts of the animal might exhibit different RNA expression patterns. Also, since the animals are settling on algae, it is interesting how contamination was avoided. Please elaborate on this. 

Are sufficient details of methods and materials provided to allow replication by others?

Partly

Is the rationale for creating the dataset(s) clearly described?

Yes

Are the datasets clearly presented in a useable and accessible format?

Yes

Are the protocols appropriate and is the work technically sound?

Yes

Reviewer Expertise:

Comparative Genomics, Comparative Developmental Biology, Phylogenetic Analyses

We confirm that we have read this submission and believe that we have an appropriate level of expertise to confirm that it is of an acceptable scientific standard, however we have significant reservations, as outlined above.
